# Association of blood pressure with decline in renal function and time until the start of renal replacement therapy in pre-dialysis patients: a cohort study

**DOI:** 10.1186/1471-2369-12-38

**Published:** 2011-08-11

**Authors:** Moniek CM de Goeij, Nora Voormolen, Nynke Halbesma, Dinanda J de Jager, Elisabeth W Boeschoten, Yvo WJ Sijpkens, Friedo W Dekker, Diana C Grootendorst

**Affiliations:** 1 Department of Clinical Epidemiology, Leiden University Medical Center, Albinusdreef 2, 2333 ZA Leiden, the Netherlands; 2 Hans Mak Institute, Koningin Wilhelminalaan 29-B, 1411 EL Naarden, the Netherlands; 3 Department of Nephrology, Leiden University Medical Center, Albinusdreef 2, 2333 ZA Leiden, the Netherlands; 4 Bronovo Hospital, Bronovolaan 5, 2597 AX the Hague, the Netherlands; 5 Linnaeus Institute, Kennemer Gasthuis, Boerhaavelaan 22, 2035 RC Haarlem, the Netherlands

**Keywords:** blood pressure, chronic kidney disease stages IV-V, estimated glomerular filtration rate, pre-dialysis care, renal replacement therapy

## Abstract

**Background:**

To investigate whether high blood pressure accelerates renal function decline in patients with advanced chronic kidney disease (CKD), we studied the association of systolic (SBP) and diastolic blood pressure (DBP) with decline in renal function and time until the start of renal replacement therapy (RRT) in patients with CKD stages IV-V on pre-dialysis care.

**Methods:**

In the PREPARE-1 cohort 547 incident pre-dialysis patients, referred as part of the usual care to outpatient clinics of eight Dutch hospitals, were included between 1999 and 2001 and followed until the start of RRT, mortality, or end of follow-up (January 1^st ^2008). Main outcomes were rate of decline in renal function, estimated as the slope of available eGFR measurements, and time until the start of RRT.

**Results:**

A total of 508 patients, 57% men and median (IQR) age of 63 (50-73) years, were available for analyses. Mean (SD) decline in renal function was 0.35 (0.75) ml/min/1.73 m^2^/month. Every 10 mmHg increase in SBP or DBP resulted in an accelerated decline in renal function (adjusted additional decline 0.04 (0.02;0.07) and 0.05 (0.00;0.11) ml/min/1.73 m^2^/month respectively) and an earlier start of RRT (adjusted HR 1.09 (1.04;1.14) and 1.16 (1.05;1.28) respectively). Furthermore, patients with SBP and DBP above the BP target goal of < 130/80 mmHg experienced a faster decline in renal function (adjusted additional decline 0.31 (0.08;0.53) ml/min/1.73 m^2^/month) and an earlier start of RRT (adjusted HR 2.08 (1.25;3.44)), compared to patients who achieved the target goal (11%). Comparing the decline in renal function and risk of starting RRT between patients with only SBP above the target (≥ 130 mmHg) and patients with both SBP and DBP below the target (< 130/80 mmHg), showed that the results were almost similar as compared to patients with both SBP and DBP above the target (adjusted additional decline 0.31 (0.04;0.58) ml/min/1.73 m^2^/month and adjusted HR 2.24 (1.26;3.97)). Therefore, it seems that especially having SBP above the target is harmful.

**Conclusions:**

In pre-dialysis patients with CKD stages IV-V, having blood pressure (especially SBP) above the target goal for CKD patients (< 130/80 mmHg) was associated with a faster decline in renal function and a later start of RRT.

## Background

Chronic kidney disease (CKD) and end-stage renal disease (ESRD) are major public health problems worldwide, because of rapidly increasing numbers of prevalent and incident cases [1-3]. The demand for pre-dialysis care is growing due to the increasing number of patients with late-stage CKD. Patients on pre-dialysis care need to be treated to slow down decline in renal function and to postpone the start of renal replacement therapy (RRT; dialysis and transplantation).

High blood pressure is an important independent predictor of decline in renal function in the general population [4] and in several subgroups [5-8]. Furthermore, high blood pressure is also a risk factor for the progression to CKD [9,10] and ESRD [11-14] in the general population. Once a person has developed early stage CKD, blood pressure has a persisting detrimental effect on decline in renal function resulting in an accelerated progression to ESRD [15-19]. However, little is known about the association of blood pressure with decline in renal function in patients with CKD stages IV-V on pre-dialysis care.

Therefore, it is important to study the association of blood pressure with progression of CKD in patients with CKD stages IV-V on pre-dialysis care. Guidelines from the Kidney Disease Outcomes Quality Initiative (K/DOQI), Seventh Report of the Joint National Committee (JNC 7), and the American Heart Association (AHA) propose a blood pressure treatment target goal of < 130/80 mmHg through all stages of CKD [20-22]. Because the use of this proposed treatment target goal of < 130/80 mmHg in pre-dialysis patients is not evidence based, it is important to investigate whether this goal is indeed beneficial in this specific population. Therefore, the aim of our study was to investigate the association of systolic (SBP) and diastolic blood pressure (DBP) with progression of CKD as assessed by decline in renal function and time until the start of RRT in patients with CKD stages IV-V on pre-dialysis care.

## Methods

### Study design and participants

The PREdialysis PAtient REcord-1 (PREPARE-1) study is a follow-up study in which consecutive incident adult patients with CKD stages IV-V were included from outpatient clinics of eight Dutch hospitals when referred for pre-dialysis care between 1999 and 2001. Patients had been referred to these outpatient clinics if creatinine clearance was below 20 ml/min. Furthermore, in these patients the need for RRT was expected within one year. Patients who spent less than one month on pre-dialysis care and patients with prior RRT were excluded. The clinical course of pre-dialysis patients was followed through the medical charts until the start of dialysis, transplantation, death, or January 1^st ^2008, whichever was earliest. Predefined data on demography, anthropometry, and clinical symptoms were extracted from medical charts at inclusion. All available data concerning laboratory measurements during pre-dialysis care were extracted from the Hospital Information Systems. The study was approved by the Institutional Review Boards of the participating hospitals and conducted in concordance with Good Clinical Practice Guidelines.

### Measurements and definitions

Blood pressure and variables used for adjustment in multivariable analyses were assessed at the first pre-dialysis visit (baseline) and measured according to the standard care applied in each individual hospital. The standard procedure for measuring blood pressure is using a device dependent on cuff occlusion of the arm, with the patient in sitting or lying position. The blood pressure treatment target goal was defined as SBP < 130 mmHg and DBP < 80 mmHg according to the K/DOQI, JNC 7, and AHA guidelines [20-22]. Estimated glomerular filtration rate (eGFR) was estimated using the abbreviated Modification of Diet in Renal Disease (MDRD) formula, taking into account age, sex, race, and serum creatinine [23]. Baseline serum creatinine measurement was defined as the measurement closest to the start of pre-dialysis care, within 90 days before and 14 days after the start.

### Outcomes

For the present analyses, the first year of pre-dialysis care was used as follow-up time, because blood pressure was only assessed at baseline and blood pressure levels can change during pre-dialysis care. Therefore, we expect that the effect of baseline blood pressure on progression of CKD weakens after several years of follow-up. Outcomes were decline in renal function and time until the start of RRT. The rate of decline in renal function was estimated by fitting a regression line through the available eGFR measurements for each individual patient. This resulted in slopes expressing the monthly loss of eGFR. eGFR measurements between one month prior to inclusion and one year after inclusion were used and at least two measurements had to be available to estimate the rate of decline. Patients with eGFR measurements only available between one month prior to inclusion and one month thereafter were excluded from the analyses for decline in renal function (n = 20). The start of RRT was defined as starting dialysis or being transplanted within the first year of pre-dialysis care.

### Statistical analyses

Continuous data were expressed as mean (standard deviation, SD) and skewed data as median (boundaries of interquartile range, IQR). Baseline characteristics were presented for the total study population and stratified for patients below or above the blood pressure treatment target goal. SBP and DBP were both analyzed in steps of 10 mmHg increase and in categories. We used clinically relevant categories for SBP (< 130 (reference group), 130-149, 150-169, 170-189, and ≥ 190 mmHg) and for DBP (< 80 (reference group), 80-89, 90-99, and ≥ 100 mmHg). The reference categories chosen were based on the treatment target goal of < 130/80 mmHg. Furthermore, the range of the SBP and DBP categories (steps of 10 and 20 mmHg respectively) were selected by using the distribution of these variables, to end up with enough power in each category. Additionally, SBP and DBP were combined based on the treatment target goal of < 130/80 mmHg. The additional effect of having DBP above 80 mmHg on top of having SBP above 130 mmHg, compared to the single effect of SBP ≥ 130 mmHg or DBP ≥ 80 mmHg, was investigated. For this analysis, patients were stratified into four categories based on the combination of SBP and DBP (< 130/< 80 (reference group), ≥ 130/< 80, < 130/≥ 80, and ≥ 130/≥ 80 mmHg).

A linear regression analysis was used to assess the association of blood pressure with decline in renal function during the first year of follow-up. Multivariable analyses were used to adjust for the possible confounders age, sex, race, smoking, primary kidney disease, presence of cardiovascular disease (CVD; angina pectoris, coronary disease, and/or myocardial infarction), and presence of diabetes mellitus (DM).

The Kaplan-Meier method was used to estimate the crude risk and a Cox proportional hazard regression analysis was used to estimate the adjusted risk of starting RRT during the first year of pre-dialysis care. The risk of starting RRT was adjusted for the same confounders used in the multivariable linear regression analyses. The time from the first pre-dialysis visit until the start of RRT or end of the first year of follow-up was used in the Cox proportional hazard regression as follow-up time. Mortality and lost to follow-up were censored events. All p-values are two-tailed and p < 0.05 was considered statistically significant. Data were analyzed with PASW/SPSS version 17.

Multiple sensitivity analyses were performed. First, sensitivity analyses were performed with only the first six months or with complete follow-up (until January 1^st ^2008) instead of the first year of follow-up, to investigate whether differences in follow-up time lead to different results. Second, a sensitivity analysis was performed with SBP and DBP stratified in quartiles and quintiles to make sure that the associations found are robust and not dependent on the choice of categories. Third, it can be argued that our analyses should be additionally adjusted for proteinuria, hemoglobin, and baseline eGFR. However, data regarding proteinuria, hemoglobin, and baseline eGFR were not available for all patients included in the analyses (n = 405, n = 444, and n = 445 respectively). Therefore, adjusting for these variables was only performed as a sensitivity analysis, in order to maintain power.

## Results

### Baseline characteristics

From the 547 incident pre-dialysis patients included, 525 patients had no prior RRT and spent more than one month on pre-dialysis care. SBP and DBP measurements at baseline were present for 508 patients and for 436 patients two or more eGFR measurements were available to estimate the rate of decline in renal function. Of these 508 patients, 89% had SBP and/or DBP above the blood pressure treatment target goal (SBP ≥ 130 mmHg and/or DBP ≥ 80 mmHg). Most of the patients on pre-dialysis care were referred by nephrologists (76%). This percentage was the same in patients below and patients above the target (78% and 76% respectively, Table 1). Patients with SBP and/or DBP above the target were older, smoked more, had a slightly higher body mass index (BMI), lower eGFR, higher levels of proteinuria, and a higher prevalence of CVD (Table 1). At baseline, patients with an available estimated rate of decline in renal function (n = 436) were slightly older, had a higher eGFR, lower levels of proteinuria, and less co-morbidities, than patients without available eGFR data (n = 72).

**Table 1:** Demographic, anthropometric, and clinical characteristics at baseline for the total study population and stratified for reaching the blood pressure treatment target goal

	All n = 508	Below target^Δ^ n = 58	Above target^Δ^ n = 450
Age (years)	63 (50-73)	55 (45-71)	64 (50-73)
Sex (% men)	57	57	56
Caucasian (%)	97	97	96
BMI (kg/m^2^)*	26 ± 5	25 ± 4	26 ± 5
Smokers/quitters < 1 year before inclusion (%)	56	50	57
Primary kidney disease (%)			
Diabetes mellitus	17	16	17
Glomerulonephritis	10	14	9
Interstitial nephritis	16	17	15
Renal vascular disease/nephrosclerosis	23	17	23
Baseline eGFR (ml/min/1.73 m^2^)^†^	13.1 ± 5.8	14.0 ± 6.0	13.0 ± 5.7
Proteinuria (g/24 h)^‡^	2.0 (0.7-3.9)	1.1 (0.6-2.9)	2.2 (0.8-4.0)
Hemoglobin (g/dl)^§^	11.3 ± 1.6	11.4 ± 1.8	11.3 ± 1.6
Co-morbidities (%)			
Anemia^·^	47	46	47
Cardiovascular disease^γ^	36	28	37
Diabetes mellitus	27	24	27
Malignancy	11	10	11
Referral to pre-dialysis care by nephrologist (%)	76	78	76

Median with boundaries of interquartile range (IQR) is given for age and proteinuria and for the other continuous variables mean ± standard deviation (SD) is given. ^Δ ^Below the target was defined as SBP < 130 and DBP < 80 mmHg and above the target as SBP ≥ 130 and/or DBP ≥ 80 mmHg. * Available for 472 patients. ^† ^estimated glomerular filtration rate (eGFR) calculated with the abbreviated Modification of Diet in Renal Disease (MDRD) formula and available for 445 patients. ^‡ ^Available for 405 patients. ^§ ^Available for 444 patients. ^· ^Defined as a hemoglobin level < 11 g/dl. ^γ ^Defined as the presence of angina pectoris, coronary disease, and/or myocardial infarction.

Of all patients, 92% were treated with anti-hypertensive drugs at baseline. Patients below the blood pressure treatment target goal were primarily prescribed angiotensin converting enzyme (ACE)-inhibitors and/or diuretics whereas patients above the target were prescribed calcium-antagonists and beta-blockers just as often (Table 2). Angiotensin II-inhibitors were prescribed the least. Furthermore, the number of anti-hypertensive drug types was higher in patients above the blood pressure treatment target goal than in patients below the target. Of the patients treated with anti-hypertensive drugs, only 11% achieved the blood pressure treatment target goal of < 130/80 mmHg.

**Table 2:** Blood pressure characteristics at baseline for the total study population and stratified for reaching the blood pressure treatment target goal

	All n = 508	Below target^Δ^ n = 58	Above target^Δ^ n = 450
Systolic blood pressure (mmHg)	152 ± 27	114 ± 12	157 ± 25
Diastolic blood pressure (mmHg)	83 ± 14	68 ± 8	85 ± 13
Anti-hypertensive drug types (%)			
ACE-inhibitor	43	60	40
Angiotensin II-inhibitor	20	10	21
Beta-blocker	39	31	40
Calcium antagonist	45	22	48
Diuretics	48	52	48
Other*	14	7	15
Number of anti-hypertensive drug types (%)			
0	8	9	8
1	26	35	24
2	28	33	28
≥ 3	38	23	40

Systolic (SBP) and diastolic blood pressure (DBP) are presented as mean ± standard deviation (SD).

^Δ ^Below the target was defined as SBP < 130 and DBP < 80 mmHg and above the target as SBP ≥ 130 and/or DBP ≥ 80 mmHg. * Defined as the prescription of alpha blocker, vasodilator, and/or central working agent.

### Decline in renal function

The median (IQR) number of available eGFR measurements during the first year of follow-up was 9 (6-13) and 98.6% of the patients had three or more eGFR measurements. In the total group of 436 patients, mean (SD) decline in renal function was 0.35 (0.75) ml/min/1.73 m^2^/month. Mean (SD) decline in renal function was not dependent on the number of eGFR measurements and was 0.37 (0.89), 0.38 (0.62), 0.41 (0.72), and 0.26 (0.71) ml/min/1.73 m^2^/month for patients with 2-6, 7-9, 10-13 and ≥ 14 eGFR measurements (quartiles) respectively. The analysis of SBP in steps of 10 mmHg showed a linear association, every 10 mmHg increase in SBP resulted in an accelerated decline in renal function (crude additional decline 0.03 (0.01;0.06) ml/min/1.73 m^2^/month, Table 3). The analysis of SBP stratified in categories showed similar results, every consecutive category above the reference category of < 130 mmHg (mean (SD) decline 0.22 (0.74) ml/min/1.73 m^2^/month) resulted in an accelerated decline in renal function (Table 3).

**Table 3:** Association of systolic and diastolic blood pressure with decline in renal function during the first year of pre-dialysis care

	n	Baseline eGFR* (ml/min/ 1.73 m^2^)	Crude additional decline (ml/min/ 1.73 m^2^/month)	Adjusted additional decline^†^ (ml/min/ 1.73 m^2^/month)	Adjusted additional decline^‡^ (ml/min/ 1.73 m^2^/month)
Systolic blood pressure (mmHg):					
Per 10	436		0.03 (0.01;0.06)	0.04 (0.02;0.07)	0.04 (0.02;0.07)
< 130	79	14.0	0^§^	0	0
130-149	116	12.8	0.13 (-0.09;0.34)	0.15 (-0.06;0.36)	0.14 (-0.07;0.35)
150-169	125	12.7	0.14 (-0.08;0.35)	0.19 (-0.02;0.39)	0.17 (-0.04;0.38)
170-189	72	14.2	0.19 (-0.05;0.43)	0.27 (0.04;0.51)	0.24 (0.00;0.48)
≥ 190	44	13.3	0.34 (0.06;0.61)	0.44 (0.16;0.71)	0.43 (0.15;0.70)
Diastolic blood pressure (mmHg):					
Per 10	436		0.07 (0.02;0.12)	0.05 (0.00;0.10)	0.05 (0.00;0.11)
< 80	122	13.4	0^γ^	0	0
80-89	151	13.7	0.08 (-0.10;0.26)	0.06 (-0.12;0.23)	0.07 (-0.11;0.25)
90-99	98	13.3	0.12 (-0.07;0.32)	0.12 (-0.08;0.31)	0.12 (-0.08;0.32)
≥ 100	65	11.9	0.29 (0.07;0.52)	0.23 (0.00;0.45)	0.22 (-0.01;0.44)
Blood pressure treatment target goal (mmHg):					
< 130/< 80	47	14.2	0^	0	0
≥ 130/< 80	75	13.0	0.21 (-0.07;0.48)	0.31 (0.04;0.58)	0.31 (0.04;0.58)
< 130/≥ 80	32	13.8	0.22 (-0.12;0.55)	0.21 (-0.12;0.54)	0.26 (-0.08;0.59)
≥ 130/≥ 80	282	13.1	0.27 (0.04;0.50)	0.30 (0.08;0.53)	0.31 (0.08;0.53)

The decline (95% confidence interval) presented for each blood pressure category indicates the additional decline in renal function compared to the reference category (systolic blood pressure (SBP) < 130 mmHg, diastolic blood pressure (DBP) < 80 mmHg, and SBP/DBP < 130/< 80 mmHg). A negative decline is equivalent to a slower decline in renal function.

* The mean estimated glomerular filtration rate (eGFR) in ml/min/1.73 m^2 ^at baseline. ^† ^Adjusted for sex and age. ^‡ ^Adjusted for sex, age, race, smoking, primary kidney disease, and the co-morbidities cardiovascular disease (CVD) and diabetes mellitus (DM). ^§ ^The mean (standard deviation, SD) decline in renal function is 0.22 (0.74) ml/min/1.73 m^2^/month. ^γ ^The mean (SD) decline in renal function is 0.26 (0.90) ml/min/1.73 m^2^/month. ^The mean (SD) decline in renal function is 0.13 (0.85) ml/min/1.73 m^2^/month.

Every 10 mmHg increase in DBP also resulted in an accelerated decline in renal function (crude additional decline 0.07 (0.02;0.12) ml/min/1.73 m^2^/month, Table 3). Furthermore, every consecutive category above the reference category of < 80 mmHg (mean (SD) decline of 0.26 (0.90) ml/min/1.73 m^2^/month) resulted in an accelerated decline in renal function. However, the most pronounced decline in renal function was present in patients with DBP ≥ 100 mmHg.

Using the blood pressure treatment target goal (SBP < 130 mmHg and DBP < 80 mmHg) showed that patients with SBP and/or DBP above the target had an accelerated decline in renal function compared to patients with SBP and DBP below the target (mean (SD) decline 0.13 (0.85) ml/min/1.73 m^2^/month). Adjustment for confounders did not change these results essentially (Table 3).

### Start of Renal Replacement Therapy

**Figure 1:**
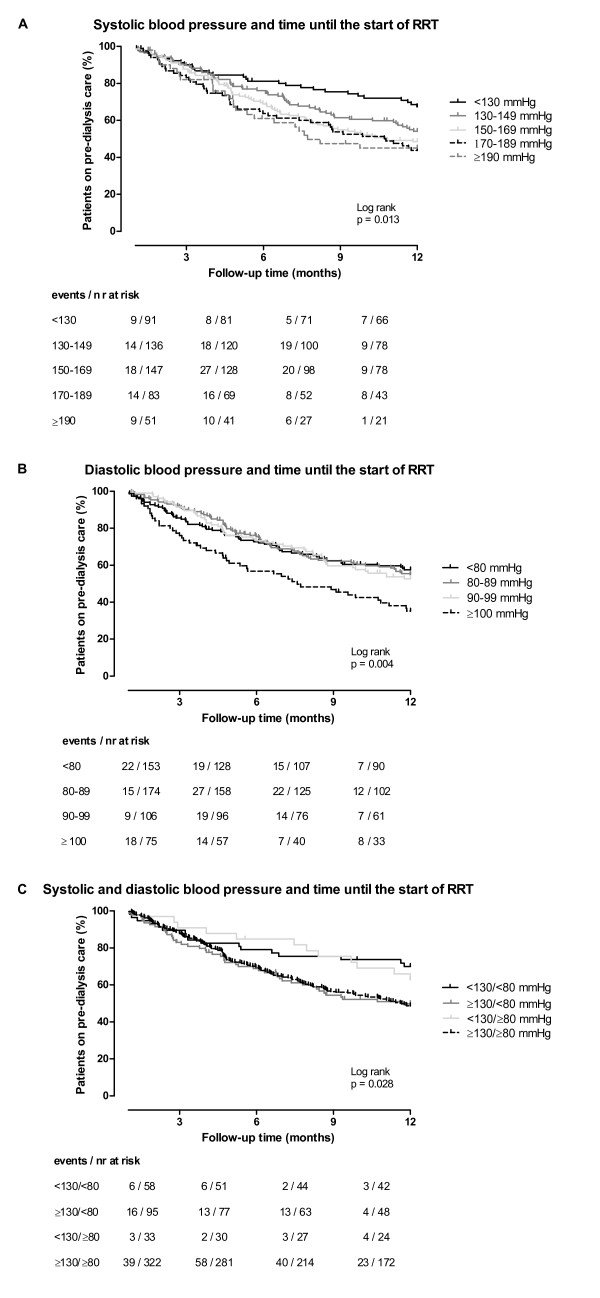
**Kaplan-Meier curve for time until the start of renal replacement therapy**. Kaplan-Meier curve for the association of systolic (SBP), diastolic blood pressure (DBP) and the combination of SBP and DBP with time until the start of renal replacement therapy (RRT) during the first year of follow-up in pre-dialysis patients. Follow-up time in months (during the first year of follow-up) on the x-axis and the percentage of patients on pre-dialysis care on the y-axis is plotted and stratified for SBP (A), DBP (B), and SBP/DBP (C) blood pressure categories. The numbers below the figures give the number of patients 'at risk' (n) and the events in each blood pressure category for 3 month intervals.

During the first year of follow-up, of all patients 23% started hemodialysis and 22% peritoneal dialysis, 1% was transplanted, 5% died, 1% was lost to follow-up while 48% were still on pre-dialysis care. Median (IQR) follow-up time was 351 (144-365) days, while follow-up time in patients starting RRT was 145 (87-234) days. In patients below and above the blood pressure treatment target goal at baseline, mean eGFR was 6.7 and 8.2 ml/min/1.73 m^2 ^at the start of RRT respectively. The crude association of SBP and DBP categories with time until the start of RRT is shown in Figure 1A and 1B respectively. Table 4 shows the crude and the adjusted hazard ratios (HRs) for every 10 mmHg increase in SBP and DBP, and for clinically relevant blood pressure categories compared to the reference category of SBP < 130 mmHg or DBP < 80 mmHg respectively. Patients with higher SBP had an earlier start of RRT. On a continuous scale, every 10 mmHg increase in SBP resulted in an earlier start of RRT (crude HR 1.08 (1.03;1.13)). Furthermore, the adjusted risk of starting RRT was 1.60 fold higher (95% CI, 1.02;2.50) in patients with SBP 130-149 mmHg compared to patients with SBP < 130 mmHg (Table 4). For DBP, every 10 mmHg increase resulted in an earlier start of RRT (crude HR 1.15 (1.04;1.27)). The analysis with categories showed that only patients with DBP ≥ 100 mmHg had a pronounced earlier start of RRT (crude HR 1.84 (1.26;2.69), Table 4 and Figure 1B).

**Table 4:** Association of systolic and diastolic blood pressure with time until the start of renal replacement therapy during the first year of pre-dialysis care

	Events/n	Crude HR	Adjusted HR^†^	Adjusted HR^‡^
Systolic blood pressure (mmHg):				
Per 10	235/508	1.08 (1.03;1.13)	1.09 (1.04;1.14)	1.09 (1.04;1.14)
< 130	29/91	1	1	1
130-149	60/136	1.52 (0.97;2.36)	1.54 (0.99;2.41)	1.60 (1.02;2.50)
150-169	74/147	1.82 (1.19;2.80)	1.88 (1.22;2.90)	1.96 (1.27;3.02)
170-189	46/83	2.09 (1.31;3.32)	2.20 (1.37;3.53)	2.25 (1.40;3.63)
≥ 190	26/51	2.10 (1.24;3.57)	2.27 (1.33;3.90)	2.21 (1.28;3.80)
Diastolic blood pressure (mmHg):				
Per 10	235/508	1.15 (1.04;1.27)	1.15 (1.04;1.27)	1.16 (1.05;1.28)
< 80	63/153	1	1	1
80-89	76/174	1.02 (0.73;1.43)	1.03 (0.74;1.44)	1.05 (0.75;1.48)
90-99	49/106	1.09 (0.75;1.58)	1.11 (0.76;1.62)	1.11 (0.76;1.62)
≥ 100	47/75	1.84 (1.26;2.69)	1.85 (1.26;2.71)	1.88 (1.27;2.78)
Blood pressure treatment target goal (mmHg):				
< 130/< 80	17/58	1	1	1
≥ 130/< 80	46/95	1.93 (1.11;3.37)	2.03 (1.15;3.57)	2.24 (1.26;3.97)
< 130/≥ 80	12/33	1.18 (0.56;2.47)	1.18 (0.56;2.47)	1.31 (0.62;2.78)
≥ 130/≥ 80	160/322	1.91 (1.16;3.16)	1.96 (1.19;3.23)	2.08 (1.25;3.44)

The hazard ratio (HR) (95% confidence interval, CI) presented for each blood pressure category indicates the increased risk of starting renal replacement therapy (RRT) compared to the reference category (systolic blood pressure (SBP) < 130 mmHg, diastolic blood pressure (DBP) < 80 mmHg, and SBP/DBP < 130/< 80 mmHg).

^† ^Adjusted for sex and age. ^‡ ^Adjusted for sex, age, race, smoking, primary kidney disease, and the co-morbidities cardiovascular disease (CVD) and diabetes mellitus (DM).

Using the blood pressure treatment target goal (SBP < 130 mmHg and DBP < 80 mmHg) showed that patients with SBP above the target and DBP below the target started RRT earlier (crude HR 1.93 (1.11;3.37)) compared to patients with both SBP and DBP below the target (Table 4 and Figure 1C). Patients with SBP < 130 mmHg (below target) and DBP ≥ 80 mmHg (above target) had a similar risk of starting RRT as the reference group (patients with SBP < 130 mmHg and DBP < 80 mmHg). Having both SBP and DBP above the treatment target goal resulted in a higher risk of starting RRT (crude HR 1.91 (1.16;3.16)) compared to having both SBP and DBP below the target. Adjustment for confounders did not change these results essentially (Table 4).

### Sensitivity analyses

To test the robustness of our findings, several sensitivity analyses were performed. First, in a sensitivity analysis with only six months of follow-up, results of the primary analyses were confirmed and point estimates were similar. Analyses with complete follow-up showed the same trend as in the primary analyses, however the associations were somewhat diluted. Every 10 mmHg increase in SBP or DBP resulted in an accelerated decline in renal function (adjusted additional decline 0.04 (0.01;0.07) and 0.05 (0.00;0.11) ml/min/1.73 m^2^/month respectively) and an earlier start of RRT (HR 1.07 (1.03;1.11) and 1.10 (1.01;1.19) respectively). Second, other choices of blood pressure categories showed the same results as found in the primary analyses. Third, when including the covariates proteinuria, hemoglobin, and baseline eGFR in the model (sample size of 404 patients), the point estimates for decline in renal function and time until the start of RRT remained similar. With every 10 mmHg increase in SBP or DBP, decline in renal function accelerates (adjusted additional decline 0.04 (0.02;0.07) and 0.07 (0.02;0.12) ml/min/1.73 m^2^/month respectively) and the start of RRT is earlier (HR 1.07 (1.01;1.13) and 1.17 (1.04;1.31) respectively).

## Discussion

The results of this study show that 89% of the patients with CKD stages IV-V starting pre-dialysis care did not reach the blood pressure treatment target goal of SBP < 130 mmHg and DBP < 80 mmHg, even though the majority of these patients (92%) were treated with antihypertensive drugs. Both higher SBP and DBP were associated with a faster progression of CKD. More specific, patients with SBP ≥ 130 mmHg or DBP ≥ 100 mmHg had an accelerated decline in renal function (eGFR) during pre-dialysis care which was accompanied by an earlier start of RRT, compared to patients with SBP < 130 mmHg or DBP < 100 mmHg. Further analysis showed that in patients with SBP above the target goal (≥ 130 mmHg), additionally having DBP above the target goal (≥ 80 mmHg) did not result in an accelerated decline in renal function or an earlier start of RRT.

Our study is the first European study to report on the association of both SBP and DBP with the progression of CKD, as assessed by decline in renal function and time until the start of RRT, in a well-defined population of pre-dialysis patients with CKD stages IV-V. Every 10 mmHg increase in SBP or DBP resulted in an accelerated decline in renal function (adjusted additional decline 0.04 (0.02;0.07) and 0.05 (0.00;0.11) ml/min/1.73 m^2^/month respectively). Our data are in line with the findings of several observational studies such as the MDRD study [24], which showed that an increased mean arterial pressure is associated with an accelerated decline in renal function in patients with an eGFR between 13 and 24 ml/min/1.73 m^2^. However, CKD patients with an eGFR below 13 ml/min/1.73 m^2 ^were excluded from the MDRD study because they were likely to start dialysis before the effect of diet and blood pressure control could be evaluated [25]. Furthermore, patients with severe co-morbidities were excluded and the patient population in the MDRD study consisted mostly of patients with polycystic kidney disease. Our patients may therefore be more representative of patient populations as seen by treating physicians in outpatient clinics, as our patient selection did not restrict on etiology of the primary kidney disease, level of eGFR or severity of co-morbidities. The results of our study also showed that every 10 mmHg increase in SBP or DBP respectively resulted in an adjusted 1.09 (95% CI 1.04;1.14) and 1.16 (95% CI 1.05;1.28) fold increased risk of starting RRT earlier. These results are in line with a study performed by Levin *et al *[26], who found that every 5 mmHg increase in SBP or DBP resulted in an earlier start of RRT (HR 1.02, 95% CI 1.00;1.04, and HR 1.05, 95% CI 1.01;1.09, respectively) in a Canadian cohort of patients on pre-dialysis care (CKD stages IV-V, n = 4,231).

Our data are not in line with some previous published interventional trials on the effect of lowering blood pressure in CKD patients [27-29]. These studies showed that in patients with CKD stages III-V intensive blood pressure lowering (< 130/80 mmHg versus < 140/90 mmHg) was not beneficial on the progression of CKD, defined as decline in renal function or time until the start of dialysis, transplantation, death and/or doubling of serum creatinine. There are several possible explanations for these contradictory findings. First, the study populations of the trials differ from our study population, because the trials mainly consist of patients with CKD stage III and our observational cohort consists of patients with advanced stages of CKD (IV-V). Furthermore, the trials apply many patient exclusion criteria that will result in a relatively more healthy study population compared to our cohort. Second, the achieved blood pressure goal in the intensively treated patients (< 130/80 mmHg) had a large overlap with the normal treated patients (< 140/90 mmHg). This finding suggests that blood pressure control is very difficult in patients with advanced stages of CKD and this can explain the high percentage (89%) of pre-dialysis patients in our cohort not reaching the treatment target goal, which is in line with another study [30]. It can be reasoned that blood pressure control is easier to achieve in patients with early stages of CKD, as shown by Nakayama *et al *[31]. Finally, the trials found that the effect of lowering blood pressure was beneficial in patients with more severe proteinuria and our cohort mainly consists of patients with proteinuria (89%).

The PREPARE-1 study has potential strengths and limitations. First, in our cohort an equal number of patients started HD and PD and therefore the results cannot be generalized to the United States pre-dialysis population. Furthermore, it has been estimated that in the general population 36-65% of the patients with an eGFR below 15 ml/min/1.73 m^2 ^are not treated by a nephrologist [32]. Therefore, perhaps our results cannot be generalized to all patients with an eGFR below 15 ml/min/1.73 m^2^, but only to those receiving pre-dialysis care. However, for clinical practice our cohort is a very representative and relevant population. Second, for estimating the rate of decline in renal function, we assumed that the decline in renal function is linear in advanced CKD stages. It has been shown previously that linearity of the course in eGFR is a plausible assumption, although on theoretical grounds over a longer period of time an exponential decline could be present [24]. Third, SBP and DBP were collected only once at the start of pre-dialysis care. This may lead to less precision and more variability of the SBP and DBP measurement at baseline, possibly resulting in underestimated effects. Furthermore, blood pressure may change during pre-dialysis care. A time-dependent analysis with time varying blood pressure would be more accurate compared to an analysis with only one blood pressure measurement at baseline. However, such data were not available in our study. As an alternative, we showed that when using follow-up of six months the association of blood pressure with decline in renal function and time until the start of RRT remained similar, whereas the results where somewhat diluted when using complete follow-up. The latter may indeed suggest that blood pressure changes over time. We chose to use the first year of follow-up in our primary analyses in order to have a more precise estimation of the association of blood pressure with decline in renal function and time until the start of RRT. Fourth, it is possible that the association of blood pressure with progression of CKD is confounded by the prognosis of patients. However, baseline characteristics showed that referral of patients is mainly done by nephrologists and this percentage is comparable between patients below and above the blood pressure treatment target goal, which may give an indication that prognosis is similar. Furthermore, adjustment for sex, age, race, smoking, primary kidney disease, co-morbidities, hemoglobin, eGFR, and proteinuria eliminated a part of this confounding effect, but we cannot exclude some residual confounding of our effect measures. Adjustment for decline in renal function prior to starting pre-dialysis care was not possible, because too few eGFR measurements were available. Fifth, the aim of this study was to investigate the association of baseline blood pressure with progression of CKD irrespective of blood pressure control by medication. Therefore, the definition of the blood pressure treatment target goal was solely based on blood pressure and results were not adjusted for the use of anti-hypertensive drugs. Finally, data regarding proteinuria, hemoglobin, and baseline eGFR were not available for all patients. Therefore, in order to maintain power and to avoid selection bias, we chose not to include these variables into our primary analyses. However, after adjustment for these covariates the point estimates for decline in renal function and time until the start of RRT remained similar.

What could be an explanation for the found association of hypertension with an accelerated decline in renal function and an earlier start of RRT? Endothelial dysfunction is a pathological consequence of hypertension [33] leading to vessel damage, causing atherosclerosis, and subsequently arterial stiffness. Arterial stiffness in turn can lead to an increased afterload, left ventricular hypertrophy, reduced coronary perfusion, congestive heart failure, and altered or reduced blood supply to tissues including the kidney [34]. In pre-dialysis patients, arterial stiffness can lead to glomerular hypertension and thereby increased glomerular permeability and excessive filtration of proteins. Protein ultrafiltrates are toxic to the proximal tubules, resulting in tubular damage and scarring. Glomerular hypertension and increased ultrafiltration of proteins contribute to the progression of chronic renal damage and thereby renal function [35]. It is also possible that blood pressure increases as the result of an accelerated decline in renal function, resulting in a vicious circle between blood pressure increase and decline in renal function. In our cohort, arterial stiffness seems a plausible explanation because with increasing pulse pressure, which is a marker for arterial stiffness, decline in renal function accelerated and time until the start of RRT decreased (data not shown).

## Conclusions

The proposed blood pressure treatment target goal for patients with CKD is < 130/80 mmHg, according to the K/DOQI, JNC 7, and AHA treatment guidelines for hypertension in CKD patients [20-22]. The results of our study show that pre-dialysis patients with blood pressure below this target have the slowest decline in renal function and the latest start of RRT. Our results support the beneficial effect of keeping blood pressure levels, especially for systolic blood pressure, below this treatment target goal at the start of pre-dialysis care. Randomized controlled trials are necessary to demonstrate whether active blood pressure lowering below this target is beneficial in pre-dialysis patients.

## Competing interests

The PREPARE-1 study is an independent academic study designed and carried out by the Department of Clinical Epidemiology from the Leiden University Medical Center in collaboration with the Hans Mak Institute (Naarden) and the participating hospitals. This study was funded by an unrestricted grant from Amgen (the Netherlands and Switzerland). The sponsor of the study was not involved in study design, collection of data, statistical analyses, interpretation of data, writing of the manuscript, or in the decision to submit the paper for publication. None of the authors have declared a conflict of interest.

## Authors' contributions

MCMdG performed the statistical analyses and drafted the manuscript. NV collected data and revised the manuscript critically. NH, DJdJ, EWB, YWJS, FWD, and DCG contributed to the conception and the design and revised the manuscript critically. All authors have given final approval for this version to be submitted.

## Pre-publication history

The pre-publication history for this paper can be accessed here:

http://www.biomedcentral.com/1471-2369/12/38/prepub
